# A Review of the Middle Ear Risk Index as a Prognostic Tool for Outcome in Middle Ear Surgery

**DOI:** 10.7759/cureus.31038

**Published:** 2022-11-03

**Authors:** Manisha Dash, Prasad Deshmukh, Sagar S Gaurkar, Ajinkya Sandbhor

**Affiliations:** 1 Otolaryngology-Head and Neck Surgery, Jawaharlal Nehru Medical College, Datta Meghe Institute of Medical Sciences, Wardha, IND; 2 Otolaryngology-Head and Neck Surgery/Surgical Oncology, Jawaharlal Nehru Medical College, Datta Meghe Institute of Medical Sciences, Wardha, IND

**Keywords:** review article, chronic otitis media (com), tympanic membrane perforation, hearing, quality of life (qol), middle ear risk index, tympanoplasty surgery

## Abstract

This review article aims to scrutinize the studies conducted to determine a relationship between preoperative Middle Ear Risk Index (MERI) factors and postoperative graft acceptance and audiological gain in patients undergoing tympanoplasty procedures in middle ear surgeries. Critical analysis is done on numerous research and types of studies that were done in this area during the past years. The clinical and technical aspects connected to disease and its care have a variety of effects on the morphological and functional outcome of tympanoplasty. A better comprehension of these characteristics aids in better disease prognostication, surgical planning, and patient counseling. At the end of this review, we can conclude that the MERI score is inversely proportional to the post-operative graft acceptance and audiological gain. The accumulated MERI is hence a good prognostic factor for the hearing outcome of surgery.

## Introduction and background

Chronic otitis media (COM), formerly known as chronic suppurative otitis media (CSOM), is a long-lasting inflammation of the middle ear and mastoid cavity that frequently causes perforation of the tympanic membrane. It is a potentially dangerous condition that can frequently result in serious damage and irreversible aftereffects, such as deadly intracranial problems, which may place an unfair load on the psychosocial status of the patient and their family. A low number of deaf persons work in managerial, technical, and professional positions because this condition restricts mobility, reduces interpersonal interactions, and has a substantial financial impact. In current society, COM and the accompanying hearing loss are major, therefore it is worthwhile to make an effort to help individuals who are affected. Because of a lack of awareness and specialized medical care, it poses a severe health hazard in developing nations like India [1]. One of the most challenging aspects of otologic surgery continues to be achieving persistent good hearing results in the presence of COM. Patients regularly have procedures to treat the disease because of the disease's high incidence. The significance of determining the disease severity and forecasting the results of surgical care is brought out by this [2].

COM causes conductive hearing loss, typically a mild conductive loss of 10 to 20 dB; while in some cases, ossicular chain erosion can occur, causing a more profound audiologic variation of 50 to 70 dB, which is usually considered a serious disability [3].

Although medical treatment can give temporary relief, surgery is the definitive option. Tympanoplasty is an operative procedure that helps in the reconstruction of the sound-conducting mechanism of the middle ear. It is a procedure aimed to prevent reinfection and restore hearing ability. This surgical technique often combined with the repair of the perforated tympanic membrane is done with/without the reconstruction of ossicles [4]. Some studies concluded that the outcome of tympanoplasty depends on various factors like size and location of the perforation, ossicular status, type of surgical technique, degree of otorrhoea, etc. [5,6]. The ability to predict the outcome of the surgical procedure prior to surgery will play a crucial role in explaining and convincing the patient for surgery, especially in developing nations where the cost of surgery is a major limitation.

The American Academy of Ophthalmology and Otolaryngology Subcommittee on Conservation of Hearing established a standard classification for surgery for chronic middle ear infection in 1965, and tympanoplasty was defined as a procedure to clear disease from the middle ear and to rebuild the hearing mechanism [7].

Kartush introduced the MERI to help with the tympanoplasty prognosis. In order to predict the success of tympanoplasty, Becvarovski and Kartush developed and updated the middle ear risk index in 2001 [8]. This index generates a numerical indicator of the severity of the middle ear disease. The recognized preoperative and intraoperative risk variables for the prognosis of tympanoplasty are combined into a quantitative value by MERI. In order to clarify these fundamental facts and divide patients into other prognostic categories, Kartush modified the Austin classification and introduced the middle ear risk index (MERI). They assigned a risk value to each of the parameters they monitored, which included otorrhea, eardrum perforation, cholesteatoma, ossicular status, middle ear granulations or effusions, prior surgery, and smoking. It also allows meaningful study comparisons by delineating essential data and stratifying cases within various prognostic categories. There are various reports discussing prognostic factors in tympano-mastoid surgery and their impact on hearing results [8]. Black introduced the system of Surgical, Prosthetic, Infection, Tissues and Eustachian tube function (SPITE), as prognostic indicators for tympanoplasty [9].

Throwing light on the various parameters which determine a successful tympanoplasty, important ones include age, gender, type and site of perforation, middle ear condition, presence or absence of middle ear granulations, cholesteatoma, drainage status of the ear at the time of surgery, co-morbidities associated with the patient (diabetic status, smoker), most of which has been combined meticulously to form the above-mentioned scoring system. 

In this review, we aimed to evaluate the role of Middle Ear Risk Indices (MERI) in predicting the outcome among patients undergoing tympanoplasty procedures through records and studies available which includes various age groups with different outcomes in order to eliminate bias.

Search strategy

**Figure 1:**
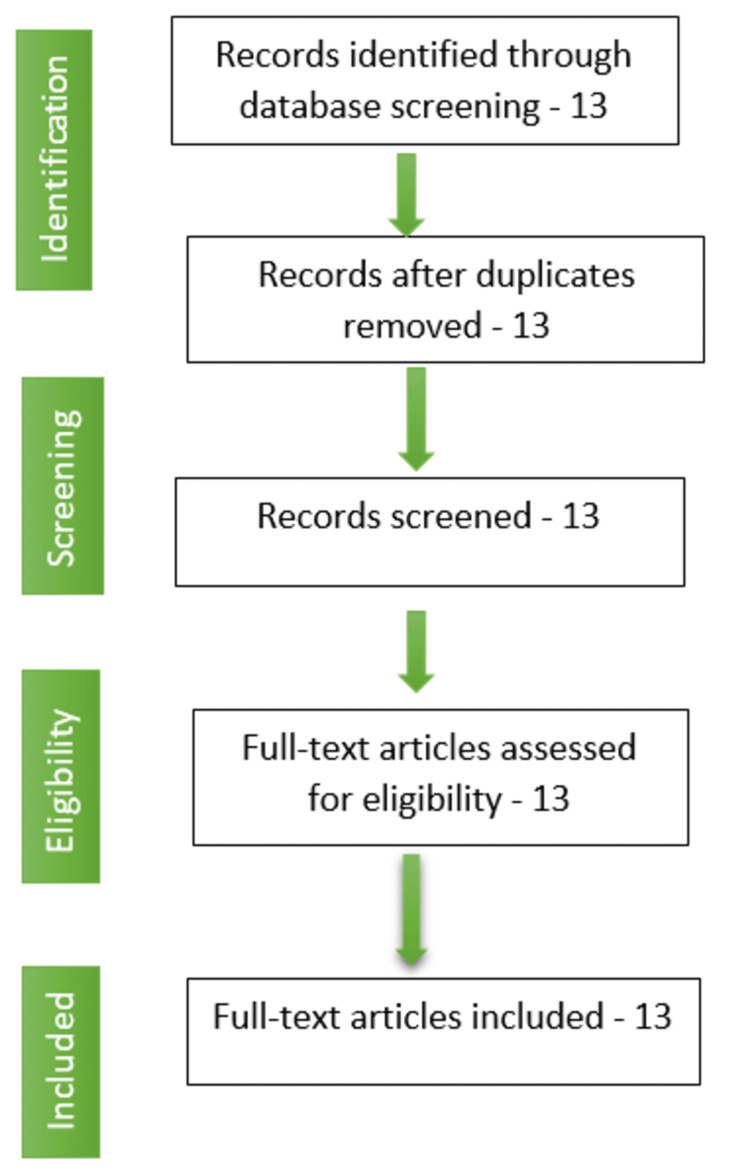
Search strategy for the review

We conducted a systematic review by searching PubMed and Google Scholar without date, geographic location of study, or language restrictions (performed January 2021). Preferred Reporting Items for Systematic Reviews and Meta-Analyses (PRISMA) guidelines were followed (Figure 1). Our search terms included: "Middle Ear Risk Index" + "Tympanoplasty" or "Chronic suppurative otitis media" or "Hearing loss" or " Graft acceptance" or “Graft acceptance”.

Selection criteria

Randomized and non-randomized controlled trials, cohort studies, and prospective studies including actual live patients were all considered when choosing the sources for the current review. Letters to the editor, case reports, technical reports, and research on animals were all eliminated from this study.

## Review

Selected articles were reviewed meticulously and observation was drawn. This review included both the adult population as well as the pediatric age group. MERI calculated was based as follows (Table 1).

**Table 1:** MERI indices A value is assigned for each risk factor, and then the values are added to determine the MERI (Middle Ear Risk Index). M –malleus, I-incus, S-stapes

RISK FACTOR	VALUE ASSIGNED RISK
Otorrhoea (Belluci)	
1-Dry	0
2-Occasionally wet	1
3-Persistently wet	2
4-Wet, cleft palate	3
Perforation	
Absent	0
Present	1
Cholesteatoma	
Absent	0
Present	2
Ossicular status (Austin/Kartush)	
0- M +I+S	0
A-M+S+	1
B-M+S-	2
C-M-S+	3
D-M-S-	4
E-Ossicle head fixation	2
F-Stapes fixation	3
Middle ear-granulation or effusion	
No	0
Yes	2
Previous surgery	
None	0
Staged	1
Revision	2
Smoker	
No	0
Yes	2

Selected articles were reviewed meticulously and observation was drawn (Table 2). This review included both the adult population as well as the pediatric age group.

**Table 2:** Inference of the various studies reviewed

S.NO	AUTHOR	TYPE OF STUDY	NO OF PATIENTS	CORRELATION OF MERI WITH	OUTCOME
1	Gundu et al. [10}	Prospective	60	Graft uptake and hearing improvement	Lower the MERI score better the outcome
2	Harugop et al. [11]	Retrospective observational	287	Graft uptake and hearing improvement	Lower the MERI score better the outcome
3	Kumar et al. [2]	Prospective	50	Pre-operative eustachian function(ETF) with graft uptake and hearing improvement	Normal eustachian tube function, better uptake of graft and lower the MERI score better the outcome
4	Sharma et al. [12]	Prospective	50	Hearing improvement in terms air-bone (A-B) gap	Lower the MERI score better the outcome of A-B gap
5	Abd ElNaem et al. [13]	Prospective	100	Graft uptake and hearing improvement	Lower the MERI score better the outcome
6	Verma et al. [14]	Prospective	200	Graft uptake and hearing improvement	Lower the MERI score better the outcome
7	Almazrou et al. [15]	Retrospective	44	Post operative hearing improvement in children	MERI was not a reliable tool for predicting the hearing results of ossicular reconstruction in children.
8	de laTorre et al. [16]	Prospective	67	Graft uptake	MERI was found to be directly proportional for tympanoplasty failure in children.
9	Pinar et al. [17]	Case series	231	Graft uptake and post operative A-B gap	High MERI had a much higher possibility of canal wall down surgery and lower chance of successful tympanoplasty.
10	Zhu et al. [18]	Retrospective cohort	385	Graft uptake and post operative A-B gap	MERI score greater than three was found to be a significant predictor of postoperative hearing
11	Kalyanasundaram and Narendran [19]	Prospective study	50	Graft uptake	MERI score has inverse correlation with graft uptake status
12	Singh et al. [20]	Prospective study	90	Graft uptake and hearing improvement	Lower the MERI, better the outcome(functional and audiological)
13	Nallapaneni et al. [21]	Prospective cohort comparative study	75	Graft uptake and pre-operative and post- operative pure tone audiometry (PTA)	Increase MERI, lowers the graft uptake, decreases hearing benefit post-operatively

Discussion

One of the most well-known and well-studied health challenges in underdeveloped countries is COM. The recovery of each patient is different, despite the fact that new surgical procedures have been created over decades and have been the focus of much research. This is due to the disease's different origins having a significant impact on both the disease's course and its treatment. Due to its great incidence, we see a lot of patients, and it takes several operations to both treat the sickness and restore hearing. In a country like India, where the cost of surgery and time away from work are major considerations, striving to establish a standard approach to foresee the outcome of surgery and counsel the patient accordingly plays a crucial role. The middle ear risk index is one of the various methods employed to predict the results of surgery. It considers both preoperative and postoperative middle ear conditions.

The prognostic factors and hearing outcomes following tympanomastoid surgery have been covered in numerous papers, with age playing a significant role. Generally speaking, tympanoplasty in children has a marginally lower success rate than in adults [22,23]. However, some writers [24-26] came to the opposite conclusion, saying that patient age had no bearing on tympanoplasty surgical success. Due to the lack of clarity surrounding the prognostic criteria that contribute to surgical success, tympanoplasty done on children has generated debate throughout the years. Because of a child's developing immune system, shorter and less effective eustachian tubes, recurring upper respiratory infections, and challenges with postoperative care, some people believe that surgery is ineffective in treating this condition [27,28].

While some otolaryngologists think dry ear has little bearing on graft success, the majority of them think it is essential. In patients who had a dry ear for three months prior to surgery, Uyar et al.'s [29] research revealed a much greater rate of graft absorption. However, several investigations [6,26] did not find a statistically significant link between dry ears and any of the studies.

An earlier study reported the condition of the contralateral ear as a predictive factor. Patients with normal opposite ears had a worse transplant success rate than healthy opposite ear patients, according to Sevil et al.'s research [30]. Between the two groups, the claimed success rates did not, however, show any discernible variations.

Pinar et al. [17] noted that the success rate of tympanoplasty was higher when the opposite ear did not have a perforation or atelectasis and came to the conclusion that canal wall-down tympanoplasty is frequently needed to eradicate the disease in patients with severe MERI.

The outcome of hearing following tympanoplasty depends on a number of well-established parameters relating to both the pathologic state and the surgical approach and method. The best tympanoplasty reconnects a large tympanic membrane with the stapes footplate via either an intact or reconstructed ossicular chain, restoring sound protection for the round window by creating a closed air-containing middle ear, and rebuilding the sound pressure transformation mechanism for the oval window.

Patients with lower MERI had considerably better pre-operative and post-operative air and bone conduction than patients with higher MERI [12]. Hearing improved by 4 to 6 dB in persons with mild MERI. The majority of the research described in the study discovered that the difference between the air bone (A-B) gap before and after surgery was statistically significant.

## Conclusions

With the goal of establishing a link between the MERI score and post-operative anatomical and audiological gain, we have endeavored to assess the diverse findings of numerous studies through this review article. It can be concluded that the MERI risk score can be used to predict the outcome of tympanoplasty. Through this review we attempt to help surgeons, predominately practicing in developing countries where monetary cringe always exists behind the screen, to counsel the patients better regarding outcomes and provide them with assurance in some form. Patients with lower MERI risk scores had better prognoses in terms of graft acceptance, improvement in hearing, and also in achieving good A-B gap closure. Patients with higher MERI risk scores had higher rates of graft rejection and no significant improvement in hearing. Although most of the studies have shown a positive outcome which mainly included the adult population but the current literature involving the pediatric age group was found to be unsatisfactory. Future studies require a larger database or population study to bridge the research gap.
